# Blend Sign and Haemorrhage Location and Volume Predict Late Recurrence and Mortality in Intracerebral Haemorrhage Patients

**DOI:** 10.3390/jcm12196131

**Published:** 2023-09-22

**Authors:** Frank Schreiber, Jan-Niklas Kuschel, Marwa Klai, Christian Chahem, Philipp Arndt, Valentina Perosa, Anne Assmann, Marc Dörner, Michael Luchtmann, Sven Günther Meuth, Stefan Vielhaber, Solveig Henneicke, Stefanie Schreiber

**Affiliations:** 1 Department of Neurology, Otto-von-Guericke University, 39120 Magdeburg, Germany; frank.schreiber@dzne.de (F.S.); janniklaskuschel@gmail.com (J.-N.K.); marwouta30@hotmail.fr (M.K.); c.chahem@gmail.com (C.C.); ph.ulbrich@web.de (P.A.); vperosa@mgh.harvard.edu (V.P.); stefan.vielhaber@med.ovgu.de (S.V.); solveig.henneicke@med.ovgu.de (S.H.); 2 German Center for Neurodegenerative Diseases (DZNE), 39120 Magdeburg, Germany; marc.doerner@usz.ch; 3 J. Philip Kistler Stroke Research Center, Massachusetts General Hospital, Boston, MA 02114, USA; 4 Department of Consultation-Liaison Psychiatry and Psychosomatic Medicine, University Hospital Zurich, University of Zurich, 8091 Zurich, Switzerland; 5 Department of Neurosurgery, Otto-von-Guericke University, 39120 Magdeburg, Germany; 6 Department of Neurology, Heinrich-Heine-University, 40225 Düsseldorf, Germany; svenguenther.meuth@med.uni-duesseldorf.de; 7 Center for Behavioral Brain Sciences (CBBS), Otto-von-Guericke University, 39106 Magdeburg, Germany

**Keywords:** computed tomography, computed tomography angiography, intracerebral haemorrhage, mortality, recurrence

## Abstract

Background: Studies on risk factors for primary intracerebral haemorrhage (ICH) focus on short-term predictive values of distinct clinical parameters or computed tomography (CT) markers and disregard the others. We, therefore, studied independent predictive values of demographic, clinical, and CT markers regarding ICH expansion, late ICH recurrence, and late mortality. Methods: In a retrospective study of 288 patients with primary ICH, ICH localization (158 lobar, 81 deep, and 49 cerebellar), volume, blend sign, spot sign, finger-like projections, and subarachnoid haemorrhages were evaluated. ICH localization-specific differences for demographic (age, sex), clinical parameters (vascular risk factors, antiplatelet, and anticoagulation therapy), and CT markers were evaluated using logistic regression. We applied Cox proportional hazards modelling using these parameters to predict risk factors for ICH expansion, late ICH recurrence, and late mortality. Results: The blend sign in lobar ICH relates to increased risk of ICH expansion (HR2.3), late ICH recurrence (HR2.3), and mortality (HR1.6). Age, conditions requiring antiplatelet medication, deep ICH localization, volume, and blend sign represented the most important independent factors impacting overall mortality. Conclusions: Blend sign at baseline ICH is a manifestation of underlying detrimental vascular processes that signal increased ICH expansion risk, although is also indicative of long-term risks for late recurrent ICH and late mortality.

## 1. Introduction

Intracerebral haemorrhages (ICHs) are a major health problem in our society. They account for 2 million (10–15%) of the 15 million strokes globally each year, with an annual incidence of 10–30 per 100,000 persons, which mainly depends on age, arterial hypertension, and the usage of antithrombotic drugs [1]. Long-term clinical outcomes, ICH recurrence rates, and mortality depend on similar variables [2,3].

Neuroimaging signs derived from computed tomography (CT) could also help in the prediction of longitudinal ICH courses, with the advantage of their investigator independence and (semi)quantitative measurability. CT signs are thereby available even in emergency settings and even with otherwise absent information regarding the patient’s medical history. Such CT signs comprise the blend sign [4], CT angiography (CTA) spot sign [5,6], finger-like projections (FLP) [7], and subarachnoid haemorrhage (SAH) [7]. The blend sign and spot sign, for example, predict a higher risk of more rapid ICH volume expansion [4,8,9], which in turn forecasts worse clinical outcomes [10].

ICH long-term prognosis further relies on ICH localization, in that, the risk of ICH volume expansion and mortality seems to be most pronounced in deep and cerebellar ICH [3,11]. Lobar ICH, on the contrary, has larger ICH baseline volumes, without such distinct expansion tendencies, but higher ICH recurrence rates [3,11].

This variety of imaging alongside clinical and demographic non-imaging-based predictors demands a careful analysis of their independent impact on the ICH’s long-term outcome, to guide clinicians in their respective meaning for the patient’s overall prognosis. Most studies on the ICH course have, thus far, however, only taken into account a restricted number of these variables, usually either focusing on non-imaging or isolated imaging markers.

To unravel the significance of non-imaging and imaging-based markers for ICH long-term prognosis together, we conducted a retrospective longitudinal study on a hospital-based cohort with lobar, deep, or cerebellar ICH. We took into account demographics, vascular risk, antithrombotic therapy, and several CT signs alongside the ICH volume to analyse their potential as predictors of ICH volume expansion, late recurrence, and late mortality.

## 2. Materials and Methods

### 2.1. Study Population

The study was designed as a retrospective cohort study. We screened the database of the Department of Neurology at Otto-von-Guericke University, Magdeburg, for patients who had an ICH and at least one CT within 24 h after ICH symptom onset. We identified *n* = 393 ICH patients who were admitted and treated in the department from 26 July 2003 to 2 June 2018.

Patients were excluded if they had a secondary ICH due to head trauma, aneurysms or vascular malformations, bleeding tendency, such as disseminated intravascular coagulation, brain infarction, or brain tumour, or because of poor scan quality. The final cohort included *n* = 288 patients.

**Figure 1:**
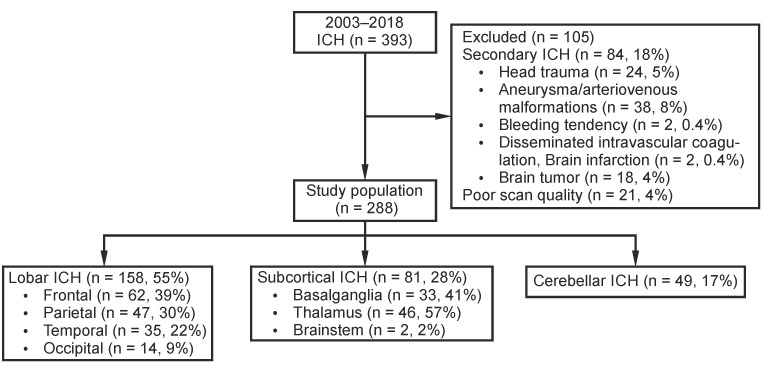
Flowchart of patient selection and classification. ICH, intracerebral haemorrhage; *n*, number.

ICH localization was classified according to the Cerebral Haemorrhage Anatomical RaTing instrument (CHARTS) as lobar (frontal, parietal, temporal, occipital, or insular, including subcortical white matter), deep (basal ganglia, thalamus, or brainstem), and infratentorial (cerebellum) [12]. Figure 1 demonstrates the patient selection and classification processes.

Medical records were retrospectively screened for cardiovascular risk factors and treatment with antithrombotic therapy, i.e., antiplatelet therapy and anticoagulation, at the time of ICH. Arterial hypertension was identified in cases where blood pressure exceeded 130/80 mmHg or through the use of antihypertensive drugs. Diabetes mellitus was diagnosed as fasting plasma glucose levels > 7.0 mmol/L, or >11.1 mmol/L two hours after a glucose tolerance test, or through the intake of antidiabetic drugs. Hyperlipidaemia was defined as abnormal blood levels of low-density lipoprotein cholesterol (>2.6 mmol/L) and/or triglycerides (>1.7 mmol/L), or through the intake of lipid-lowering medication. Smoking was considered evident if a patient’s total consumption reached a minimum of 5 pack-years.

Information on the existence or absence of each vascular risk factor was available in *n* = 206 (72%) patients, while in the remainder (*n* = 82, 28%) there was missing information on at least one risk factor. We, thus, created a vascular risk factor score (VRFS) to mitigate this absence, summing the ascertainable risks by counting each risk factor as one point, and dividing by the number of available risk factors (e.g., a score of 2 points/3 available risk factors = 0.67).

Information on the intake of any antithrombotic therapy was available in *n* = 222 (77%). Patients received either antiplatelet therapy only (*n* = 37, 17%), anticoagulative therapy only (*n* = 49, 22%), a combination therapy (*n* = 44, 20%), or no antithrombotic therapy (*n* = 92, 41%).

### 2.2. Protocol Approvals and Data Availability

The Clinical Ethics Committee in the medical faculty of Otto-von-Guericke University approved this retrospective study (No. 28/16) and waived the need for patient consent. Informed consent was obtained from all subjects contacted in this study to evaluate longitudinal progression. The study was performed in accordance with the relevant local and national guidelines and regulations. Grouped data will be shared as anonymized following a request from a qualified investigator.

### 2.3. Longitudinal Data

The ICH date was considered as a baseline, and ICH volume expansion was assessed by follow-up CTs at baseline during the patient’s hospital stay (see below). Patients were followed longitudinally, with a median (range) follow-up time of 4.7 (1.0 15.1) years, to assess ICH recurrence and mortality after baseline. Longitudinal follow-up was conducted by evaluating all available medical documentation (including from transferring and subsequent physicians and care providers), and/or telephone or in-person interview of the patient and/or their relative(s). Each interview was performed by a trained senior neurologist (S.S.). In cases where there was no contact information, mortality was inquired about through the patient’s registration office. In total, data on ICH recurrence was available for 243 patients, while survival could be established for 224 patients.

We separately considered early and late ICH recurrence and mortality, respectively. Based on recent studies, median hospitalization after ICH accounted for around 10 days [13]. Thus, we defined early events as having occurred within 10 days after baseline, while late events occurred later than 10 days after baseline.

### 2.4. CT Acquisition and Analysis

Baseline CT and, if available, baseline CTA were performed through the emergency diagnostic work-up using a Somatom Definition AS (Siemens Healthineers, Erlangen, Germany) scanner. CT slice thickness was 2 mm. Baseline CTA was available in *n* = 87 (30%) patients, while *n* = 82 (29%) patients had only one baseline CT scan, *n* = 114 (40%) had two baseline CT scans, and *n* = 92 (32%) had more than two baseline CT scans. The median (range) timespan between the first and last available baseline CT was 24 h (12; 504).

Assessment of the presence of various CT signs, i.e., blend sign, spot sign, FLP, or SAH of ICH localization, as well as ICH volume and volume expansion was performed by one trained investigator (J.N.K.), taking account of all available baseline CT scans per patient, while blinded to the patient’s demographics and medical records. The choice of those CT signs was based on their suggested impact on ICH long-term outcomes [4,6,7,14].

**Figure 2:**
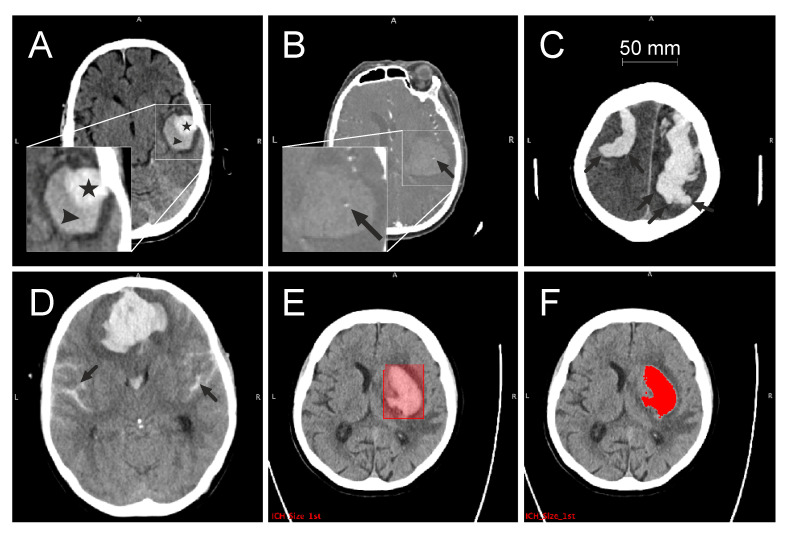
CT signs and workflow. Axial images (**A**–**D**) show different CT markers: blend sign (**A**) with two different areas of attenuation (triangle, star), spot sign (**B**) with the focus of contrast pooling (arrow), finger-like projections (**C**) with extensions longer than wide (arrows) and (**D**) subarachnoid haemorrhage (arrows). Volume quantification with the cuboid bounding region of interest (**E**) and derived ICH volume (**F**) after thresholding. CT, computed tomography; ICH, intracerebral haemorrhage.

The blend sign was defined as a blend of a relatively hyperattenuated region with an adjacent hypoattenuated area within the ICH, with a well-defined margin and a minimum density difference of at least 18 Hounsfield units (HU) between these two regions [4] (Figure 2A). The spot sign was defined as at least one focus of contrast pooling within the ICH, with a minimum density of 120 HU (Figure 2B). FLP were considered as elongated extensions arising from the ICH with a shape that is longer than it is wide, regardless of whether it reaches the cortex or not [7] (Figure 2C). SAH appears as a high-attenuating, blood-filled subarachnoid space (Figure 2D).

The attenuation of ICH is usually higher, around 56 HU, whereas the density of the surrounding tissue is lower (grey matter: 37–41 HU; white matter: 30–34 HU) [1]. Density differences were used to manually draw a bounding box containing each ICH as a region of interest (ROI) (Figure 2E). For voxels contained in this cuboid ROI, an intensity threshold was calculated using Otsu’s method to separate the hyperintense ICH volume from the normo- and hypointense surrounding tissue. After a visual inspection of segmentation results (JNK), minor threshold readjustments were carried out, when necessary, to ensure consistency. The sizes of the segmented ICH were subsequently extracted (Figure 2F).

ICH volume expansion between the first and last available baseline CT scan was considered existent in cases of a total ICH volume increase of 6 cm^3^ or a relative ICH volume increase of 33% [14]. The total ICH volume increase was applied in larger ICH, while the relative ICH volume increase was considered in smaller ICH, as has been recommended in previous studies [14].

For CT analyses, Mango software (Mango v4.0.1, UT Health San Antonio, San Antonio, TX, USA) was used and all available baseline CT and CTA slices were considered.

### 2.5. Statistical Analysis

The distribution of the ICH volumes presented an approximately log-normal distribution and was consequently log_10_ transformed for statistical analysis.

Binary variables were summarized using counts (percentages), while continuous variables were summarized using mean (SD) or median (range) values, as appropriate. For the assessment of Gaussian distribution, the Shapiro–Wilk test was applied.

A logistic regression analysis was performed to test the direct and interaction effects between the single/dual administration of antiplatelet and anticoagulative medication, and the longitudinal outcome variables (ICH volume expansion, ICH recurrence, and mortality). While significant direct effects were found between anticoagulation therapy and ICH recurrence, as well as between antiplatelet therapy and mortality, no significant interaction between antiplatelet and anticoagulation therapies, with regard to the studied outcome variables, was observed. Therefore, antiplatelet and anticoagulation therapies were treated as two independent variables in subsequent analyses; patients receiving a combination therapy were, thus, rated positive in both variables.

For group comparisons between lobar, deep, and cerebellar ICH, either univariate linear or logistic regression analysis with post hoc pairwise comparison was conducted. *p*-values ≤ 0.001 were considered significant after Bonferroni adjustment.

Significant variables derived from univariate analysis were entered into a multivariate logistic regression, *p*-values ≤ 0.05/9 = 0.005 were deemed significant.

Co-occurrence of CT signs and independence of baseline and recurrent ICH localizations were tested using Pearson’s X^2^ test, regarding *p* ≤ 0.05/4 = 0.0125 (Bonferroni adjusted) as statistically significant for co-occurrence between the four signs and *p* ≤ 0.05 for recurrence localizations.

Uni- and multivariate Cox proportional hazard (CHP) regression analyses were conducted to calculate the impact of the CT signs, ICH volume, and ICH localization, as well as the non-imaging variables baseline age, VRFS, and antiplatelet and anticoagulation therapies as independent variables on the occurrence of (i) ICH volume expansion (within hours), (ii) ICH recurrence (within years), and (iii) mortality (within years), as respective dependent variables. For the prediction of volume expansion, the ICH volume and CT signs that were measured in the first baseline scans were used, while the prediction of recurrence and mortality was based on the volume and CT signs of the final baseline scan (after possible ICH expansion).

ICH localization was dummy coded using two binary variables, one for deep and one for cerebellar location; hazard ratios for these variables are consequently relative to the lobar reference.

Peduzzi et al. recommended maintaining ratios of at least 10:1 between the number of observed events and the number of independent covariates in the CPH modelling to avoid biased estimates [15]. Correspondingly, we limited the number of independent variables in the multivariate models according to the number of events, with full datasets to six for ICH expansion, four for ICH recurrence, and nine for mortality, which were chosen by forward selection.

Bonferroni correction was applied by adjusting for the number of similar models and describing identical outputs; consequently, for the univariate CPH *p*-values ≤ 0.05/12 = 0.004 were considered significant, while *p*-values ≤ 0.05 were deemed significant for the multivariate CPH models.

Due to marked differences between patients receiving a CTA and those without (Table S1), and a high absence of the spot sign, it was not included in the multivariate CPH models.

The validity of the proportional hazard assumption was verified by testing the relationship between the corresponding set of Schoenfeld residuals and time, which was non-significant for all independent variables in the CPH models.

Analyses were performed using Statistics Toolbox in MATLAB R2021a (MathWorks, Natick, MA, USA).

## 3. Results

### 3.1. Baseline Data

#### 3.1.1. Comparison of Demographics and CT Markers

Demographics, VFRS, antithrombotic therapy, and prevalence of CT measures are presented in Table 1.

**Figure 3:**
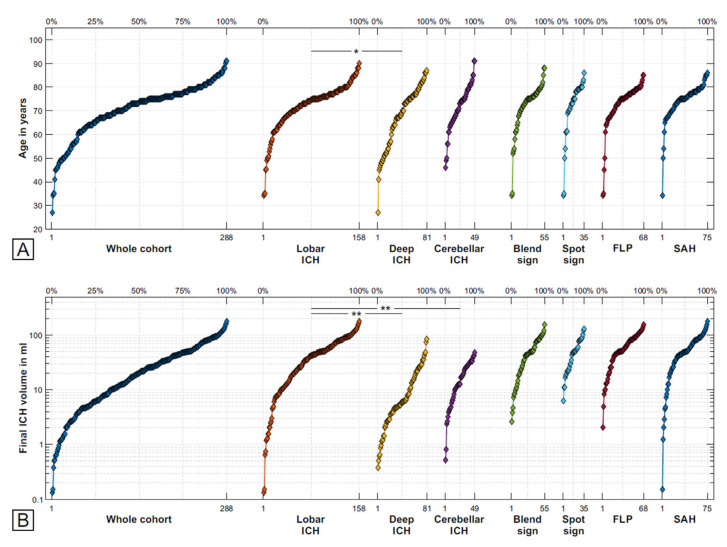
Distribution of age and final ICH volumes. Quantile plot showing the cumulative distribution of age (**A**) and final ICH volume (**B**) for the whole cohort (left), as well as the subcohorts with lobar, deep, and cerebellar ICH (centre) and carriers of the computed tomography (CT) signs blend sign, spot sign, FLP, and SAH (right). Patient indices are presented on the respective bottom axis, group percentage axis is on top. ICH volume is plotted in logarithmic scale to reflect the orders of magnitude spanned by the variable. * *p* < 0.05, ** *p* < 0.001. FLP, finger-like projections; ICH, intracerebral haemorrhage; SAH, subarachnoid haemorrhage.

Of the *n* = 288 patients, *n* = 158 (55%) had an ICH located in the lobar regions, *n* = 81 (28%) in deep, and *n* = 49 (17%) in a cerebellar location. There was a trend towards a significant difference in the age distribution between these groups (Figure 3A). No sex-based differences were present.

There were significant group differences for antiplatelet and anticoagulation therapies, blend sign, FLP, and SAH: patients with lobar ICH showed more frequent intake of antithrombotic therapy than those with deep ICH, while CT signs were most prevalent in patients with lobar ICH. The latter also showed the largest initial baseline and final baseline ICH volumes (Figure 3B).

Applying a multivariate analysis, group differences remained significant for the blend sign, FLP, SAH, and baseline and final ICH volumes.

#### 3.1.2. CT Sign Carriers

All CT signs predominantly occurred in patients with lobar ICH (Table 1), with 97% of the blend signs, 73% of the spot signs, 95% of the FLP, and 84% of SAH (Table S2).

The presence of blend sign, spot sign, and FLP was associated with larger ICH volumes, with the largest half of the ICH volumes carrying 73% of the blend signs, 84% of the spot signs, and 85% of FLP (Figure 3B). In contrast, SAH was found throughout the range of ICH volumes (range 0.1–180 mL) (Figure 3B).

A total of 71 (25%) of all patients had at least one CT sign at their initial scan, *n* = 43 (61%) of those displayed two or more, and *n* = 12 (17%) three or more. There was a significant correlation between FLP and the other studied CT signs, with SAH (X^2^ test *p* < 0.001), with blend sign (X^2^ test *p* = 0.005), and with spot sign (X^2^ test *p* = 0.028) (Table S2). The number of found CT signs correlated significantly with the ICH volume (rho = 0.56, *p* < 0.001).

### 3.2. Longitudinal Data

#### 3.2.1. ICH Volume Expansion

A total of 206 (72%) patients had at least two CT baseline scans, thereby allowing for the assessment of ICH volume expansion, which became evident in *n* = 68 (33%) cases. Patients with deep ICH underwent serial CT scanning more frequently compared to those with a lobar or cerebellar ICH (98% vs. 60%; B(1) = 3.3, *p* < 0.001; vs. 67%; B(1) = 2.9, *p* < 0.001; univariate logistic regression analysis with post hoc pairwise testing). The median time between the baseline CT scan and the first follow-up scan, which established a volume expansion, was 1 day. Median expansion volume was 6.8 mL, with a median volume increase of 58%.

**Figure 4:**
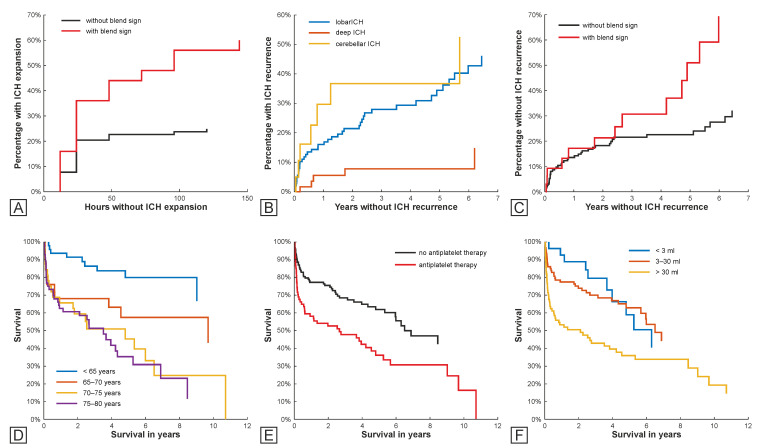
Kaplan–Meier plots for associations between predictors and outcome variables. Association between blend sign in baseline computed tomography (CT) and consequent ICH expansion (**A**); relation between ICH location (**B**), and final ICH volume (**C**) with the ICH recurrence. Influence of age (**D**), antiplatelet therapy (**E**), and final ICH volume (**F**) on overall survival. ICH, intracerebral haemorrhage.

CPH modelling revealed a significantly increased risk for ICH expansion in persons displaying the blend sign in their first baseline CT, in the univariate analysis (HR 2.64, 95% CI (1.475–4.729)), as well as in the multivariate modelling, thereby accounting for the presence of additional CT markers and non-imaging variables (HR 2.29 (1.199–4.354), Table 2A, Figure 4A). A subgroup analysis that evaluated patients with a lobar and deep ICH separately established that this finding is driven by the patients with a lobar ICH at the baseline (HR 2.80 (1.394–5.624), Table S3).

#### 3.2.2. ICH Recurrence

Information on ICH recurrence was available in *n* = 247 (86%) patients, of whom *n* = 58 (20%) had a recurrent ICH. Only *n* = 4 patients had early ICH recurrence; therefore, all further analyses took account of the patients with late ICH recurrence only. The median (range) timespan between baseline and late ICH recurrence was 9.7 (0.6; 78.4) months.

A total of 48 (20%) of those patients returned with recurrent ICH and underwent CT scans, which provided information on its localization. Recurrent ICH location was highly dependent on the localization of the baseline ICH (X^2^ test *p* < 0.001). In 41 (85%) of the patients with recurrent ICH, its location was identical to the baseline ICH. In patients with an initial lobar ICH, 92% of the recurrent ICH again occurred in lobar territories. In patients with a deep ICH at baseline, 75% of the recurrent ICH were in deep brain territories, while patients with initial cerebellar haemorrhages showed the highest variability in recurrence location, with only 57% again occurring in the cerebellar region (Table S5).

Prevalence of late ICH recurrence was 28% in lobar, 7% in deep, and 33% in cerebellar baseline ICH, thereby resulting in significant group differences (Table 1).

In univariate and multivariate CPH modelling, a deep ICH location was associated with a decreased risk (HR 0.22 (0.088–0.556) and HR 0.19 (0.064–0.570)) of ICH recurrence (Table 2B, Figure 4B). Applying univariate subgroup CPH modelling revealed that in patients with deep baseline ICH, anticoagulation therapy was related to a marked increase in ICH recurrence risk (HR 20.27 (2.062–199.325), Table S4).

In multivariate CPH modelling, carriers of the blend sign were found to be at increased risk of ICH recurrence (HR2.31 (1.171–4.539), Figure 4C), a finding driven by the subcohort with lobar ICH (HR2.30 (1.151–4.585), Table S3). ICH volume was found to be associated with decreased risk of ICH recurrence in the whole cohort (HR0.56 (0.340–0.929)), which was again driven by the lobar subcohort (HR0.57 (0.335–0.984), Table S3).

#### 3.2.3. Mortality

Information on mortality was available in *n* = 231 (80%) patients, of whom *n* = 124 (54%) had died. Early mortality occurred in only 7 patients; thus, all further analyses took account of the patients with late mortality only (*n* = 117, 52%). The median (range) timespan between baseline ICH and late mortality was 6.5 years (0.1; 10.9).

There were no significant group differences between lobar, deep, and cerebellar baseline ICH concerning late mortality (Table 1).

Univariate CPH modelling identified age, large ICH volume, and comorbidity necessitating the administration of antiplatelet medication as predictors for overall survival (OS) after baseline ICH (HR 1.06 (1.035–1.080), HR 1.96 (1.377–2.788), and HR 2.09 (1.404–3.106)). In a multivariate CPH model, these three covariates, along with the presence of a blend sign and a deep baseline ICH location emerged as the strongest predictors (age HR 1.06 (1.036–1.089), Figure 4D; antiplatelet medication HR 2.12 (1.358–3.314), Figure 4E; ICH volume HR1.95 (1.182–3.206), Figure 4F; blend sign HR 1.62 (1.030–2.539), deep location HR 2.12 (1.168–3.836), Table 2C). In subgroup analyses of the lobar subcohort, age and a condition requiring antiplatelet medication emerged as important mortality predictors in multivariate CPH modelling (HR 1.07 (1.034–1.108); HR 1.76 (1.066–2.920), Table S4), while the deep cohort age and ICH volume were the most important (HR 1.07 (1.030–1.112); HR 3.95 (1.742–8.960), Table S5).

## 4. Discussion

Here we investigated the potential of several CT signs, comprising blend sign, spot sign, FLP, and SAH, as well as ICH location and ICH volume as independent predictors of short- and long-term outcomes of primary ICH patients. We confirm that the presence of a blend sign in lobar baseline ICH relates to increased risks of ICH expansion. Moreover, it also indicates higher risks of late recurrence and mortality. The probability of late ICH recurrence was lower in deep baseline ICH, and this lower risk was mitigated by the intake of anticoagulation therapy. Localization of baseline and recurrent ICH were highly correlated, suggesting a common aetiology. Age, a condition requiring antiplatelet medication, a deep baseline ICH location, the presence of a blend sign, and ICH volume represented the most important independent risks impacting overall mortality.

We studied the presence and predictive power of multiple CT signs to account for other risk factors in a multivariate longitudinal modelling approach for long-term outcomes, while the majority of available studies focus on a single CT sign only, mostly in multi- or univariate logistic regression models for ICH expansion, functional outcome, early recurrence, and early mortality.

In the presented cohort, only a minority (25%) presented with at least one of the studied CT signs. Conversely, within these CT sign carriers, we found a high rate of co-occurrence of the studied markers, with the majority (61%) of these patients displaying at least two. Similar rates have also been observed in another study [16], suggesting that published reports concentrating on only a single one of those CT markers may be critically re-evaluated with regard to whether and how they account for the presence of multiple CT markers.

We additionally point out that since the blend sign, FLP, and spot sign are evaluated by studying the texture within the depiction of the ICH, the ICH volume necessarily has to be sufficiently large, a relation also confirmed by van Etten et al. [17]. Therefore, positivity in any of those signs is also a proxy of a larger ICH volume.

Conversely, the presence of SAH is determined by texture changes in the tissue surrounding the ICH, thereby explaining why SAH could be found throughout the range of ICH volumes, although studies advise interpreting it with caution when studying smaller-sized lobar ICH [17].

In the studied cohort, the patients undergoing CTA were a distinct subgroup with larger ICH volumes mostly in the lobar regions, but with a higher rate of antiplatelet medication. This finding is consistent with large international studies, which showed that less than 20% of ICH patients receive CTA within 8 h from symptom onset [18], thereby emphasizing the need to study non-contrast CT signs. Therefore, this difference in group composition has to be considered when attempting to compare the predictive values of the spot sign with other signs found in a general cohort of spontaneous ICH.

Most studies describe CT signs as resulting from different stages of blood coagulation in intracerebral haemorrhages, whereby hypodensities on the CT appear to correspond to blood at an early evolution stage, whereas hyperdensities represent clotted blood [4,6]. In a non-contrast CT, liquid blood tends to hypoattenuate relative to the surrounding structures in acute phases [19]. Thus, the blend sign describes the juxtaposition of blood accumulations at different stages of coagulation [4], while the spot sign and finger-like projections describe active extravasations of blood [7,20].

We point out that the presence of a blend sign in lobar ICH relates to increased risks of ICH expansion, thereby confirming previous findings [4,14,20,21]. The spot sign has been suggested as another strong predictor [20], but only reaches trend-level significance in univariate CPH analysis, likely due to the limited availability of CTA datasets. The frequency of ICH expansion was only marginally different between lobar and deep ICH groups (25% vs. 28%), therefore, suggested that higher frequencies in deep ICH could not be confirmed [11].

The blend sign has been suggested as an independent indicator of a higher early ICH recurrence risk [22]; we point out that blend sign carriers also possess increased risks for late ICH recurrence and higher mortality. We also observed that blend sign carriers have a high probability of possessing FLP and spot signs, all of which have been associated with the presence of CAA in previous studies [7,23]. CAA is known to lead to vascular fragility, associated micro- and macrobleeds, and ICH recurrence [24]. Supporting this hypothesis, blend sign and FLP occur predominantly in lobar localization in our analysis, which is also characteristic of the presence of CAA [25]. Consequently, we were able to show that recurrent ICH tended to reoccur in the same area as the initial ICH and that the risk of late ICH recurrence is notably lower in patients with deep ICH locations.

Seiffge et al. emphasize that ICH under anticoagulation is likely facilitated by an underlying high small vessel disease burden [26]. Consequently, we found that anticoagulation therapy significantly increases the risk of recurrent ICH in patients with deep ICH.

Our data confirm the important interplay of ICH location, ICH volume, and mortality [2]. Patients with deep ICH appear particularly impacted by larger ICH volumes, not only early after the ICH [11] but also with a greatly increased risk of late mortality.

The independent mortality risk posed by conditions requiring antiplatelet therapy indicates an underlying vessel disease with elevated mortality risk.

The FLP and the blend sign both signal a higher mortality risk in univariate modelling, but when adjusting for each other’s presence and evaluating their differential effect in a multivariate approach, the blend sign emerges as a more promising predictor. To further untangle the effects and possible interactions of these and other CT markers, a large number, not only of observed cases but of observed events will be necessary. The high rate of co-occurrence between the CT sign further limits the ability to resolve these questions by meta-analyses that report on only one specific marker in each study and neglect the possible presence of others. More likely, these research questions can only be answered by large multicentric cohorts, which allow the analysis of these markers and their interaction with primary data.

Due to the fact that we are a highly specialized tertiary care facility with a focus on the vascular field, there was a concentration on particularly severe cases. The fact that patients were recruited exclusively from the database of a neurological department and its intensive care stroke unit, possibly led to an exclusion of the most severe cases that had been directly admitted to the department of neurosurgery, without a previous or subsequent stay at the neurological department. While the exclusively neurosurgical patients have a substantially worse prognosis and might have been mostly excluded by the recruitment and outcome criteria, this fact might, nonetheless, have introduced a bias towards smaller ICH and better early and long-term survival.

The strengths of this study are the inclusion of a larger sample size compared to other monocentric studies and its comparative approach between lobar, subcortical, and cerebellar haemorrhages, combined with the assessment of independent risk factors in a longitudinal approach.

## 5. Conclusions

CT markers at baseline ICH are manifestations of underlying detrimental vascular processes that contribute to both the baseline ICH itself and an increase in ICH expansion risk, yet are also indicative of long-term risks for late recurrent ICH and late death, which have to be taken into account in patient risk management.

## Figures and Tables

**Table 1:** Comparison of demographics, CT markers, and outcome variables in lobar, deep, and cerebellar ICH. Unless otherwise reported mean (SD) or median (range) is given. Percentage given in relation to count within (sub)cohort. Group comparisons between lobar, deep, and cerebellar ICH were conducted by applying linear or logistic regression analysis, each with post hoc pairwise group comparisons. For univariate and multivariate regression analyses, the *p*-value for the contribution of ICH location to the regression model of the respective variable is given in the multivariate models, while accounting for the input variables (age, sex, VRFS, antiplatelet, and anticoagulation therapy). ICH volumes were log_10_ transformed for the analysis. *p*-values ≤ 0.001 were considered significant for univariate regression analysis and post hoc pairwise comparisons, while *p*-values ≤ 0.05/9 = 0.005 were deemed significant for the multivariate regression analysis. Significant results are marked in bold. ICH expansion comprises events occurring within seven days after the initial ICH. ICH recurrence and death refer to event occurrence at least 10 days after baseline ICH. CTA, computed tomography angiography; FLP, finger-like projections; ICH, intracerebral haemorrhage; *n*, number of patients with a full dataset; OR, odds ratio; SAH, subarachnoid haemorrhage; VRFS, vascular risk factor score.

Variable	Total (n = 288)	Lobar (n = 158)	Deep (n = 81)	Cerebellar (n = 49)	Group Univariate Analysis	Group Multivariate Analysis	Lobar vs. Deep	Lobar vs. Cerebellar	Deep vs. Cerebellar
**Age (years)**	70.6 (11.0)	71.9 (10.0)	67.4 (12.8)	71.5 (10.0)	*p* = 0.009		*p* = 0.003 B(1) = −4.5 (−7.4; −1.5)	*p* = 0.781 B(1) = −0.5 (−3.7; 2.8)	*p* = 0.062 B(1) = 4.0 (−0.2; 8.2)
**Male sex** ***n* (%)**	150 (52)	77 (49)	49 (62)	24 (49)	*p* = 0.100		*p* = 0.038 OR = 1.8 (1.0; 3.1)	*p* = 0.853 OR = 1.1 (0.6; 2.0)	*p* = 0.156 OR = 0.6 (0.3; 1.2)
**VRFS**	0.49 (0.27)	0.45 (0.30)	0.56 (0.20)	0.55 (0.23)	*p* = 0.012		*p* = 0.004 B(1) = 0.1 (0.0; 0.2)	*p* = 0.250 B(1) = 0.1 (−0.1; 0.3)	*p* = 0.904 B(1) = 0.0 (−0.1; 0.1)
**Antiplatelet therapy**	81 (36)	60 (45)	16 (21)	5 (42)	***p* < 0.001**		***p* < 0.001** **OR = 0.3** **(0.2–0.6)**	*p* = 0.801 OR = 0.9 (0.3–2.8)	*p* = 0.117 OR = 2.8 (0.8–9.9)
**Anticoagulation therapy**	93 (42)	76 (58)	14 (18)	3 (25)	***p* < 0.001**		***p* < 0.001** **OR = 0.2** **(0.1–0.3)**	*p* = 0.042 OR = 0.2 (0.1–0.9)	*p* = 0.563 OR = 1.5 (0.4–6.4)
**Blend sign (final)** ***n* (%)**	55 (19)	48 (30)	6 (7)	1 (2)	***p* < 0.001**	***p* < 0.001**	***p* < 0.001** **OR = 0.2** **(0.1–0.5)**	*p* = 0.003 OR = 0.0 (0.0–0.4)	*p* = 0.220 OR = 0.3 (0.0–2.2)
**CTA spot sign (final)** ***n* (%)**	35 (14)	25 (19)	7 (9)	3 (7)	*p* = 0.053	*p* = 0.088	*p* = 0.066 OR = 0.4 (0.2–1.1)	*p* = 0.090 OR = 0.3 (0.1–1.2)	*p* = 0.730 OR = 0.8 (0.2–3.2)
**FLP (final)** ***n* (%)**	68 (24)	65 (41)	3 (4)	0 (0)	***p* < 0.001**	***p* < 0.001**	***p* < 0.001** **OR = 0.1** **(0.0–0.2)**		
**SAH (final)** ***n* (%)**	75 (26)	62 (39)	4 (5)	9 (18)	***p* < 0.001**	***p* < 0.001**	***p* < 0.001** **OR = 0.1** **(0.0–0.2)**	*p* = 0.009 OR = 0.3 (0.2–0.8)	*p* = 0.020 OR = 4.3 (1.3–14.9)
**Initial baseline ICH volume (mL)**	28.7 (0.1; 180.8)	41.8 (0.1; 180.8)	9.2 (0.2; 85)	16.7 (0.5; 48.4)	***p* < 0.001**	***p* < 0.001**	***p* < 0.001** **B(1) = −32.6** **(−40.7; −24,6)**	***p* < 0.001** **B(1) = −25.0** **(−35.2; −14.8)**	*p* = 0.001 B(1) = 7.6 (3.2; 12.0)
**Final baseline ICH volume (mL)**	32.5 (0.1; 180.8)	47.1 (0.1; 180.8)	12.7 (0.4; 85.2)	17.7 (0.5; 48.4)	***p* < 0.001**	***p* < 0.001**	***p* < 0.001** **B(1) = −34.4** **(−43.2; 25.7)**	***p* < 0.001** **B(1) = −29.4** **(−40.4; −18.4)**	*p* = 0.060 B(1) = 5.0 (−0.2; −10.2)
**ICH volume expansion** ***n* (%)**	68 (24)	40 (25)	23 (28)	5 (10)	*p* = 0.029	*p* = 0.464	*p* = 0.609 OR = 1.2 (0.6–2.1)	*p* = 0.031 OR = 0.3 (0.1–0.9)	*p* = 0.019 OR = 0.3 (0.1–0.8)
**ICH recurrence** ***n* (%)**	54 (22)	42 (28)	5 (7)	7 (33)	***p* < 0.001**	***p* = 0.004**	***p* = 0.001** **OR = 0.2** **(0.1–0.5)**	*p* = 0.588 OR = 1.3 (0.5–3.5)	*p* = 0.004 OR = 6.5 (1.8–23.5)
**Mortality** ***n* (%)**	117 (52)	79 (56)	29 (43)	9 (64)	*p* = 0.136	*p* = 0.831	*p* = 0.079 OR = 0.6 (0.3–1.1)	*p* = 0.535 OR = 1.4 (0.5–4.5)	*p* = 0.147 OR = 2.4 (0.7–8.0)

**Table 2:** Cox proportional hazard modelling for outcome variables. Cox proportional hazard (CPH) regression analyses for baseline computed tomography (CT) markers and covariates as independent variables on the occurrence of (A) ICH volume expansion (within hours), (B) ICH recurrence (within years), and (C) mortality (within years), as respective dependent variables. For the prediction of volume expansion, the ICH volume and CT signs measured in initial baseline scans were used, while the prediction of recurrence and mortality was based on volume and CT signs of the final baseline scans (after possible ICH expansion). ICH location was coded using deep and cerebellar location, calculated HR are, therefore, relative to the lobar reference group. ICH volumes were log_10_ transformed. The independent variables for multivariate modelling were chosen by the forward selection, and the number of variables was determined to maintain an events-per-variable ratio ≥ 10. For univariate CPH models, Bonferroni-adjusted *p*-values ≤ 0.05/12 = 0.004 were considered significant, while *p*-values ≤ 0.05 were deemed significant for the multivariate CPH models. Significant results are marked in bold. ICH recurrence and death refer to event occurrence at least 10 days after baseline ICH. CI, confidence interval; CTA, computed tomography angiography; e, number of events with full data in the model; FLP, finger-like projections; HR, hazard ratio; ICH, intracerebral haemorrhage; *n*, number of patients with a full dataset; SAH, subarachnoid haemorrhage; VRFS, vascular risk factor score.

	Univariate Analysis				Multivariate Analysis			
Covariate	e/*n*	HR [exp(b_i_)]	95% CI	*p*-Value	e/*n*	HR [exp(b_i_)]	95% CI	*p*-Value
**(A) ICH Expansion**								
Age (a)	60/206	1.020	(0.996–1.045)	0.096	62/206	1.017	(0.992–1.042)	0.191
Male sex	60/206	1.142	(0.689–1.891)	0.607	62/206	1.113	(0.662–1.871)	0.686
VRFS	53/169	1.053	(0.380–2.920)	0.920				
Antiplatelet	50/161	1.121	(0.636–1.978)	0.693				
Anticoagulation	50/161	1.276	(0.733–2.221)	0.389				
Deep ICH	60/206	0.880	(0.523–1.481)	0.631				
Cerebellar ICH	60/206	0.442	(0.177–1.103)	0.080	62/206	0.531	(0.210–1.344)	0.182
Blend sign	**60/206**	**2.641**	**(1.475–4.729)**	**0.001**	**62/206**	**2.285**	**(1.199–4.354)**	**0.012**
CTA spot sign	19/57	2.397	(0.971–5.920)	0.058				
FLP	60/206	1.597	(0.850–2.999)	0.146				
SAH	60/206	1.396	(0.780–2.496)	0.261	62/206	1.234	(0.662–2.300)	0.509
ICH volume	60/206	1.147	(0.754–1.745)	0.521	62/206	0.915	(0.586–1.428)	0.694
**(B) ICH Recurrence**								
Age (a)	54/243	1.016	(0.991–1.041)	0.205				
Male sex	54/243	1.108	(0.646–1.900)	0.710				
VRFS	43/214	0.438	(0.146–1.313)	0.141	43/214	0.501	(0.167–1.500)	0.217
Antiplatelet	39/204	1.193	(0.620–2.296)	0.598				
Anticoagulation	39/204	1.801	(0.953–3.405)	0.070				
Deep ICH	**54/243**	**0.221**	**(0.088–0.556)**	**0.001**	**43/214**	**0.191**	**(0.064–0.570)**	**0.003**
Cerebellar ICH	54/243	1.887	(0.852–4.179)	0.118				
Blend sign	54/243	1.874	(1.028–3.417)	0.040	**43/214**	**2.305**	**(1.171–4.539)**	**0.016**
CTA spot sign	17/73	0.436	(0.136–1.403)	0.164				
FLP	54/243	1.006	(0.530–1.912)	0.985				
SAH	54/243	0.933	(0.491–1.773)	0.832				
ICH volume	54/243	1.014	(0.664–1.548)	0.950	**43/214**	**0.562**	**(0.340–0.929)**	**0.025**
**(C) Overall Survival**								
Age (a)	**117/224**	**1.057**	**(1.035–1.080)**	**<0.001**	**97/194**	**1.062**	**(1.036–1.089)**	**<0.001**
Male sex	117/224	0.804	(0.559–1.157)	0.240				
VRFS	104/205	0.710	(0.355–1.420)	0.333	97/194	0.565	(0.240–1.329)	0.191
Antiplatelet	**98/197**	**2.088**	**(1.404–3.106)**	**<0.001**	**97/194**	**2.122**	**(1.358–3.314)**	**0.001**
Anticoagulation	98/197	1.018	(0.678–1.528)	0.930	97/194	0.916	(0.586–1.431)	0.698
Deep ICH	117/224	0.728	(0.478–1.110)	0.140	**97/194**	**2.117**	**(1.168–3.836)**	**0.013**
Cerebellar ICH	117/224	1.428	(0.722–2.826)	0.306	97/194	1.432	(0.477–4.302)	0.522
Blend sign	117/224	1.684	(1.103–2.570)	0.016	**97/194**	**1.617**	**(1.030–2.539)**	**0.037**
CTA spot sign	37/66	1.356	(0.781–2.353)	0.280				
FLP	117/224	1.580	(1.062–2.349)	0.024	97/194	1.351	(0.813–2.247)	0.246
SAH	117/224	1.164	(0.778–1.743)	0.460				
ICH volume	**117/224**	**1.959**	**(1.377–2.788)**	**<0.001**	**97/194**	**1.947**	**(1.182–3.206)**	**0.009**

## Data Availability

All custom scripts and all relevant data of this study are available from the corresponding author upon reasonable request.

## Supplementary Materials

The following supporting information can be downloaded at: https://www.mdpi.com/article/10.3390/jcm12196131/s1, Table S1. Characteristics of patients with and without CTA; Table S2. Locations and co-occurrence of the CT markers; Table S3. Cox proportional hazard regression analyses for patients with lobar ICH; Table S4. Cox proportional hazard regression analyses for patients with deep ICH; Table S5. Relationship between location of baseline and recurrent ICH.
