# Supplementary material for: Blend Sign and Haemorrhage Location and Volume Predict Late Recurrence and Mortality in Intracerebral Haemorrhage Patients
Source: J Clin Med. 2023 Sep 22;12(19):6131. doi: 10.3390/jcm12196131 (PMC10573360; doi:10.3390/jcm12196131)
Supplement: Supplementary file 1 [file jcm-12-06131-s001.zip › jcm-2576633-supplementary.pdf]

Journal of
Clinical Medicine

MDPI

Blend Sign and Haemorrhage Location and Volume Predict Late Recurrence and Mortality in Intracerebral Haemorrhage Patients

Frank Schreiber [1,2,†], Jan-Niklas Kuschel [1,†], Marwa Klai [1], Christian Chahem [1], Philipp Arndt [1,2], Valentina Perosa [1,3], Anne Assmann [1], Marc Dörner [2,4], Michael Luchtmann [5], Sven Günther Meuth [6], Stefan Vielhaber [1,7], Solveig Henneicke [1,2] and Stefanie Schreiber [1,2,7,*]

[1] Department of Neurology, Otto-von-Guericke University, 39120 Magdeburg, Germany; frank.schreiber@dzne.de (F.S.); janniklaskuschel@gmail.com (J.-N.K.); marwouta30@hotmail.fr (M.K.); c.chahem@gmail.com (C.C.); ph.ulbrich@web.de (P.A.); vperosa@mgh.harvard.edu (V.P.); anne.assmann@haldensleben.ameos.de (A.A.); stefan.vielhaber@med.ovgu.de (S.V.); solveig.henneicke@med.ovgu.de (S.H.)
[2] German Center for Neurodegenerative Diseases (DZNE), 39120 Magdeburg, Germany; marc.doerner@usz.ch
[3] J. Philip Kistler Stroke Research Center, Massachusetts General Hospital, Boston, MA 02114, USA
[4] Department of Consultation-Liaison Psychiatry and Psychosomatic Medicine, University Hospital Zurich, University of Zurich, 8091 Zurich, Switzerland
[5] Department of Neurosurgery, Otto-von-Guericke University, 39120 Magdeburg, Germany;
luchtmann@gmail.com
[6] Department of Neurology, Heinrich-Heine-University, 40225 Düsseldorf, Germany; svenguenther.meuth@med.uni-duesseldorf.de
[7] Center for Behavioral Brain Sciences (CBBS), Otto-von-Guericke University, 39106 Magdeburg, Germany
* Correspondence: stefanie.schreiber@med.ovgu.de; Tel.: +49-391-67- 13431
† These authors contributed equally to this work.

Manuscript classification: Original Article

Supplemental Material

Corresponding author:

Stefanie Schreiber
Department of Neurology,
Otto-von-Guericke University
Leipziger Straße 44
39120 Magdeburg, Germany
stefanie.schreiber@med.ovgu.de

Online supplement

Table S1. Characteristics of patients with and without CTA

	CTA<br>n = 88	No CTA<br>n = 200	p-value[1]
Age	70.7 (10.5)	70.5 (11.2)	0.919
Male sex	51.1%	52%	0.893
Diabetes mellitus	17.1%	25.3%	0.175
Smoking	41.8%	37.7%	0.248
Dyslipidaemia	54.7%	58.8%	0.580
Hypertension	67.7%	82.6%	**0.012**
VRFS	44.4	50.7	0.109
Antiplatelet	47.7%	31.9%	**0.026**
Anticoagulation	49.2%	38.9%	0.154
Transferred patient	5.7%	7.0%	0.678
Blend sign	18.2%	10.5%	0.073
CTA spot sign	37.5%	Na	
FLP	26.1%	21.0%	0.337
SAH	26.1%	23.5%	0.631
No other CT sign	54.6%	62.5%	0.465
ICH baseline volume	*26.1 ml*	*11.6 ml*	**<0.001**
ICH expansion	33.3%	28.9%	0.531
ICH expansion time	*24 h*	*24 h*	0.239
ICH recurrence	23.3%	21.8%	0.794
ICH recurrence time	*470 d*	*240 d*	0.874
Mortality	56.1%	50.6%	0.458
Survival time	*136 d*	*224 d*	0.417
ICH location lobar	69.3%	48.5%	**0.001**
ICH location deep	12.9%	42.6%	**<0.001**
ICH location cerebellar	22.8%	24.2%	0.814

[1] Student's t-test for normally distributed continuous variables, Mann-Whitney-U-test for non-normally distributed continuous variables, X^2-test for categorial variables

Median values for non-normally distributed variables in cursive font.

ICH, intracerebral haemorrhage; CTA, computed tomography angiography; FLP, finger-like projections; VRFS, vascular risk factor score; SAH, subarachnoid haemorrhage

Table S2. Locations and co-occurrence of the CT markers

	Lobar	Deep	Cerebellar	Blend sign	CTA spot sign	FLP	SAH
**Lobar**	158			**36/158 (22.8%)**	24/158 (15.2%)	**62/158 (39.2 %)**	**59/158 (37.3 %)**
**Deep**		81		**1/81 (1.2%)**	6/81 (7.4%)	**3/81 (3.7 %)**	**2/81 (2.5 %)**
**Cerebellar**			49	**0/49 (0.0%)**	3/49 (6.1%)	**0/49 (0.0 %)**	**9/49 (18.4 %)**
**Blend sign**	**36/37 (97.3%)**	**1/37 (2.7%)**	**0/37 (0.0%)**	37	7/37 (18.9%)	**15/37 (40.5 %)**	12/37 (32.4 %)
**CTA spot sign**	24/33 (72.7%)	6/33 (18.2%)	3/33 (9.1%)	7/33 (21.2%)	33	**13/33 (39.4 %)**	12/33 (36.4 %)
**FLP**	**62/65 (95.4%)**	**3/65 (4.6%)**	**0/65 (0.0%)**	**15/65 (23.1%)**	**13/65 (20.0%)**	65	**38/65 (58.5 %)**
**SAH**	**59/70 (84.3%)**	**2/70 (2.9%)**	**9/70 (12.9%)**	12/70 (17.1%)	12/70 (17.1%)	**38/70 (54.3 %)**	70

Co-occurrence of the CT markers, with values indicating the fraction (percent) of cases in which the column variable was positive in the carriers of a positive row variable. For example, the bottom left entry indicates that of all patients with a subarachnoid haemorrhage 84.3% had their ICH occur in the lobar region. Using a X^2-test for independence, the variable pairs for which the null hypothesis that they are independent has to be rejected at $p < 0.05$ are marked bold.

Table S3. Cox proportional hazard regression analyses for patients with lobar ICH

Lobar subcohort	Univariate analysis				Multivariate analysis			
**Covariate**	**e/n**	**HR [exp(b_i)]**	**95% CI**	**p-value**	**e/n**	**HR [exp(b_i)]**	**95% CI**	**p-value**
**(A) ICH Expansion**								
Age [a]	34/94	1.001	(0.967–1.036)	0.937				
Male sex	34/94	0.686	(0.348–1.350)	0.275	34/94	0.542	(0.269–1.091)	0.086
VRFS	32/87	1.547	(0.485–4.934)	0.461				
Antiplatelet	29/78	0.995	(0.480–2.062)	0.990				
Anticoagulation	29/78	0.813	(0.391–1.690)	0.579				
Blend sign	34/94	2.742	(1.390–5.408)	0.004	**34/94**	**2.799**	**(1.394–5.624)**	**0.004**
CTA spot sign	12/35	1.356	(0.408–4.507)	0.619				
FLP	34/94	1.343	(0.654–2.756)	0.421				
SAH	34/94	1.419	(0.717–2.810)	0.315				
ICH volume	34/94	1.660	(0.838–3.286)	0.146	34/94	1.620	(0.736–3.566)	0.231
**(B) ICH recurrence**								
Age [a]	42/152	1.029	(0.995–1.064)	0.098				
Male sex	42/152	1.106	(0.604–2.028)	0.743				
VRFS	37/139	0.501	(0.169–1.487)	0.213	37/139	0.393	(0.125–1.236)	0.110
Antiplatelet	33/127	0.855	(0.425–1.720)	0.660				
Anticoagulation	33/127	0.741	(0.373–1.475)	0.394				
Blend sign	42/152	1.559	(0.826–2.943)	0.171	**37/139**	**2.297**	**(1.151–4.585)**	**0.018**
CTA spot sign	10/59	0.386	(0.118–1.260)	0.115				
FLP	42/152	0.749	(0.383–1.464)	0.398				
SAH	42/152	0.625	(0.314–1.244)	0.181				
ICH volume	42/152	0.671	(0.422–1.065)	0.090	**37/139**	**0.574**	**(0.335–0.984)**	**0.044**
**(C) Overall survival**								
Age [a]	**79/142**	**1.055**	**(1.025–1.087)**	**<0.001**	**67/123**	**1.070**	**(1.034–1.108)**	**<0.001**
Male sex	79/142	0.966	(0.620–1.505)	0.878	67/123	1.369	(0.799–2.345)	0.253
VRFS	73/133	0.836	(0.393–1.777)	0.642				
Antiplatelet	67/123	1.730	(1.067–2.805)	0.026	**67/123**	**1.764**	**(1.066–2.920)**	**0.027**
Anticoagulation	67/123	0.705	(0.433–1.147)	0.159				

Blend sign	79/142	1.589	(0.985–2.564)	0.058	67/123	1.502	(0.889–2.536)	0.129
CTA spot sign	23/53	0.892	(0.440–1.807)	0.750				
FLP	79/142	1.603	(1.024–2.510)	0.039	67/123	1.656	(0.945–2.903)	0.078
SAH	79/142	1.023	(0.649–1.612)	0.922				
ICH volume	79/142	1.217	(0.787–1.882)	0.378	67/123	1.696	(0.907–3.169)	0.098

Cox proportional hazard (CPH) regression analyses for patients with lobar ICH for baseline computed tomography (CT) markers and covariates as independent variables on the occurrence of (A) ICH volume expansion (within hours), (B) ICH recurrence (within years) and (C) mortality (within years) as respective dependent variable. For the prediction of volume expansion the ICH volume and CT signs measured in initial baseline scans were used, while the prediction of recurrence and mortality was based on volume and CT signs of the final baseline scans (after possible ICH expansion).
ICH location was coded using deep and cerebellar location, calculated HR are therefore relative to lobar reference group. ICH volumes were log10 transformed. The independent variables for multivariate modelling were chosen by forward selection, the number of variables was determined to maintain an events-per-variable ratio ≥ 10. For univariate CPH models Bonferroni-adjusted p-values ≤ 0.05/10 = 0.005 were considered significant, while p-values ≤ 0.05 were deemed significant for the multivariate CPH models. Significant results are marked in bold.
ICH, intracerebral haemorrhage; HR, hazard ratio; CI, confidence interval; CTA, computed tomography angiography; FLP, finger-like projections; VRFS, vascular risk factor score; SAH, subarachnoid haemorrhage; n, number of patients with full dataset; e, number of events with full data in model

Table S4. Cox proportional hazard regression analyses for patients with deep ICH

**Deep subcohort**		**Univariate analysis**				**Multivariate analysis**		
**Covariate**	**e/n**	**HR [exp(b$_i$)]**	**95% CI**	**p-value**	**e/n**	**HR [exp(b$_i$)]**	**95% CI**	**p-value**
**(A) ICH expansion**								
Age [a]	21/79	1.021	(0.984–1.058)	0.270				
Male sex	21/79	2.172	(0.795–5.930)	0.130	20/76	1.982	(0.711–5.530)	0.191
VRFS	20/75	0.928	(0.100–8.633)	0.948				
Antiplatelet	20/76	0.953	(0.319–2.852)	0.932				
Anticoagulation	20/76	2.155	(0.827–5.615)	0.116	20/76	1.906	(0.722–5.029)	0.193
ICH volume	21/79	0.549	(0.236–1.280)	0.165				
**(B) ICH recurrence**								
Age [a]	5/70	0.954	(0.897–1.016)	0.142				
Male sex	5/70	na	na	na				
VRFS	4/65	7.949	(0.037–1695.923)	0.449				
Antiplatelet	4/67	na	na	na				
Anticoagulation	**4/67**	**20.271**	**(2.062–199.325)**	**0.010**				
ICH volume	5/70	0.54	(0.089–3.276)	0.503				
**(C) Overall survival**								
Age [a]	**29/68**	**1.067**	**(1.029–1.108)**	**0.001**	**29/68**	**1.070**	**(1.030–1.112)**	**0.001**
Male sex	29/68	0.527	(0.253–1.099)	0.088				
VRFS	27/64	0.779	(0.115–5.267)	0.798				
Antiplatelet	27/66	1.942	(0.848–4.447)	0.116				
Anticoagulation	27/66	2.078	(0.907–4.762)	0.084				
ICH volume	**29/68**	**3.619**	**(1.597–8.202)**	**0.002**	**29/68**	**3.950**	**(1.742–8.960)**	**0.001**

Cox proportional hazard (CPH) regression analyses for patients with deep ICH for baseline computed tomography (CT) markers and covariates as independent variables on the occurrence of (A) ICH volume expansion (within hours), (B) ICH recurrence (within years) and (C) mortality (within years) as respective dependent variable. CT signs are left out of this subanalysis due to their scarcity in deep locations (see Table 1). For the prediction of volume expansion the ICH volume measured in initial baseline scans was used, while the prediction of recurrence and mortality was based on volume of the final baseline scans (after possible ICH expansion).

ICH location was coded using deep and cerebellar location, calculated HR are therefore relative to lobar reference group. ICH volumes were log_{10} transformed. The independent variables for multivariate modelling were chosen by forward selection, the number of variables was determined to maintain an events-per-variable ratio ≥ 10. For univariate CPH models Bonferroni-adjusted p-values ≤ 0.05/6 = 0.008 were considered significant, while p-values ≤ 0.05 were deemed significant for the multivariate CPH models. Significant results are marked in bold.

ICH, intracerebral haemorrhage; HR, hazard ratio; CI, confidence interval; CTA, computed tomography angiography; FLP, finger-like projections; VRFS, vascular risk factor score; SAH, subarachnoid haemorrhage; n, number of patients with full dataset; e, number of events with full data in model; na, not available

Table S5. Relationship between location of baseline and recurrent ICH

**Baseline↓ Recurrent→**	Lobar	Deep	Cerebellum
Lobar	34 (92 %)	1 (3 %)	2 (5 %)
Deep	1 (25 %)	3 (75 %)	0 (0 %)
Cerebellum	2 (29 %)	1 (14 %)	4 (57 %)
